# Impression Management in the Job Interview: An Effective Way of Mitigating Discrimination against Older Applicants?

**DOI:** 10.3389/fpsyg.2017.00770

**Published:** 2017-05-16

**Authors:** Irina Gioaba, Franciska Krings

**Affiliations:** Department of Organizational Behavior, Faculty of Business and Economics, University of LausanneLausanne, Switzerland

**Keywords:** age discrimination, age stereotypes, older employees, impression management, employment interview

## Abstract

The increasingly aging population in most industrialized societies, coupled with the rather age-diverse current workforce makes discrimination against older employees a prevalent issue, especially in employment contexts. This renders research on ways for reducing this type of discrimination a particularly pressing concern. Drawing on theories of social identity and impression management, our research examines the role of impression management, aimed at refuting common older worker stereotypes, in diminishing bias against older job applicants during the job interview. The study consisted in an experimental hiring simulation conducted on a sample of 515 undergraduate students. Results show that older applicants who used impression management to contradict common older worker stereotypes were perceived as more hirable than those who did not. However, despite this positive effect, discrimination persisted: older applicants were consistently rated as less hirable than their younger counterparts when displaying the same IM behavior. Taken together, this research demonstrates that older job seekers can indeed ameliorate biased interview outcomes by engaging in impression management targeting common age stereotypes; however, it also shows that this strategy is insufficient for overcoming age discrimination entirely. The current study has important implications for theory, by expanding research on the use of impression management in mitigating age discrimination, as well as for practice, by offering older employees a hands-on strategy to reduce bias and stereotyping against them.

## Introduction

The age composition of the current workforce is changing. The percentage of 55–65 year olds in the workplace has significantly increased from 16 to 20% in the years 2000–2015 and is expected to reach 21% by 2020 (Fotakis and Peschner, 2015). These changes in the demographic landscape of the modern labor market have contributed to the formation of a more diverse age workforce composition than ever before (Toossi, 2009).

In this context, the question of how to establish equal opportunities for both younger and older employees becomes of utmost importance. From personnel selection decisions and promotion opportunities to training recommendations, ample empirical evidence shows that older employees are subject to discrimination based on their age (Gordon and Arvey, 2004; Bal et al., 2011). Discrimination against older employees at employment is particularly prevalent, and it has severe negative consequences for its targets as well as for the local economy (e.g., Australian Human Rights Commission, 2015).

In their attempts to explain why age discrimination is so prevalent, scholars often identify older worker stereotypes and prejudice as the main culprit (Perry et al., 1996; Shore and Goldberg, 2005). Older worker stereotypes are predominantly negative beliefs about older employees’ competence, abilities, and efficiency. Indeed, older employees are generally perceived as being less competent, slower, less flexible, more resistant to change, less trainable, and less adaptable than younger employees (for a review, see Posthuma and Campion, 2009). Although most of these stereotypes are false (Ng and Feldman, 2012) they persist and can severely undermine older individuals’ attempts to secure employment (Krings et al., 2011).

Discrimination against older individuals in the employment context is a pressing issue and considerable efforts have been made to better understand its complexities. However, most of this body of research has focused on its prevalence and contextual determinants, such as, organizational culture (Diekman and Hirnisey, 2007) or decision makers’ prejudice (Perry et al., 1996). Much less attention has been dedicated to the actions older applicants themselves can take to reduce discrimination against them. In fact, individuals who experience discrimination are not merely passive recipients of prejudicial treatment but also agents who actively manage their experiences (Neel et al., 2013; Shih et al., 2013). Thus, applicants who are likely targets of discrimination can engage in proactive actions in order to deal with potential discriminatory experiences in selection.

However, very little is known about the effectiveness of such actions for older applicants. This is the starting point of the present study. Drawing on theorizing in the field of impression management (IM) and social identity (Roberts, 2005; Houston and Grandey, 2013), the current research examines, for the first time, the extent to which older applicants can actively manage and ultimately reduce prejudicial treatment, by employing specific IM behaviors during the job interview. IM describes individuals’ conscious or unconscious attempts to influence their image during social interactions (Gilmore et al., 1999).

### Impression Management as a Way to Reduce Age Discrimination

Many countries have instituted laws to protect individuals against age discrimination [e.g., Age Discrimination in Employment Act (ADEA) of 1967 in US; the Framework Directive 2000/78 in the European Union; Age Discrimination Act 2004 in Australia, etc.]. Apart from governmental policies and lawful actions, organizations independently undertake measures to eradicate discrimination based on age (Walker, 1999). Many of these initiatives aim at reducing bias and stereotyping of decision makers (e.g., through diversity training) or to implement selection tools that are less prone to age bias (e.g., selection tests that depend less on speed).

Attempts to combat discrimination are not confined to governmental and organizational actions only. In fact, minority group members are much concerned themselves with mitigating discrimination against their groups (Neel et al., 2013). For example, empirical evidence has documented women’s efforts to counteract gender discrimination at work by distancing themselves from traditional gender stereotypes (Heilman and Martell, 1986; Schmitt et al., 2003; Heilman et al., 2004). Another stream of research has noted how employees with disabilties manage their stigmas in order to minimze the prejudice and bias that confront them (Hebl and Kleck, 2002; Hebl and Skorinko, 2005; Jones and King, 2013; Lyons et al., 2016). Thus, minority individuals often attempt to proactively manage the (prejudicial) impressions they create in others and mitigate their consequences. The fact that individuals try to create or alter other people’s image of themselves, in the service of their personal or social goals, has long been acknowledged by research on IM. The process through which individuals attempt to control the impressions others form of them is generally referred to as IM (Leary and Kowalski, 1990). By using IM in early selection stages, individuals can create, maintain or alter a desired image of themselves (Bolino et al., 2008). For example, during a selection interview for a highly desirable job, applicants who want to elicit perceptions of ability in a future employer can do so by presenting their past accomplishments.

To obtain the desired image, individuals employ a multitude of IM behaviors. These behaviors include verbal behaviors, non-verbal behaviors, and artifactual displays (Schneider, 1981). One of the most widely used taxonomies classifies IM behaviors based on the attributions sought from the target and has identified five commonly used tactics: ingratiation, self-promotion, exemplification, supplication, and intimidation (Jones and Pittman, 1982). Integrating these early models, Tedeschi and Melburg (1984) classify IM behaviors into two categories of tactics: assertive tactics - intended to create favorable impressions - and defensive tactics - intended to repair or protect one’s image. Other conceptualizations differentiate between self-focused and others-focused IM tactics (Kacmar et al., 1992) or between honest and deceptive IM (Levashina and Campion, 2006). Of particular relevance for this study is self-promotion, which is a form of honest assertive self-focused IM. It refers to the individuals’ attempts to communicate abilities and accomplishments to appear competent (Bolino et al., 2008). Since self-promotion points to augumenting one’s status and attractivenes, it might prove a viable solution for minority group members to convey positive impressions during job interviews. Indeed, prior empirical evidence has shown that women who use self-promotion can enhance their competence ratings (Rudman, 1998).

In early selection stages and particularly during job interviews, IM is very frequently used (Ellis et al., 2002; Levashina et al., 2014) and ample evidence shows that it leads to positive outcomes, such as increased chances of being hired (McFarland et al., 2003; Van Iddekinge et al., 2007). Although to a lesser extent, the use of IM goes beyond the interview situation, and previous research has documented a weaker but postive relationship between employee IM and job performance ratings (Barrick et al., 2009). Additional research has pinpointed the mediating mechanisms between IM and interview outcomes, suggesting that different IM behaviors influence these outcomes through different paths. Specifically, others-focused tactics, such an ingratiation, have been related to increased liking and higher levels of person-organization fit, while self-focused tactics, such as self-promotion, have been linked to increased competence perceptions and higher levels of perceived job-fit (Kristof-Brown et al., 2002; Higgins and Judge, 2004; Proost et al., 2010).

However prevalent in the workplace, little is known about the effectiveness of IM for mitigating workplace discrimination. Given the core purpose of IM, namely creating a desired image of the self by influencing others’ perceptions, we posit that IM may be a particularly valuable tool minority groups members could use to actively overcome stereotypes related to their competence. Recent theorizing linking social-identity theory and IM suggests that indeed certain forms of IM, namely those grounded in social identity and group membership, may be effective in reducing discrimination, as we further outline in the following section.

### Impression Management Based on Social Identity

Identity is a prominent driving factor of many behaviors, including work-related behaviors (Miscenko and Day, 2016). In her model of professional image construction, Roberts (2005) draws on social-identity theory and IM theories to highlight the role of personal characteristics and group affiliations. The model posits that the professional image one desires to construct contains a component that is grounded in one’s social identity. This component is “shaped by one’s hopes to be publicly affiliated with or distanced from the stereotypical characteristics of the social-identity groups to which he or she belongs” (Roberts, 2005; p. 688). As a consequence, individuals engage in IM behaviors that are related to their social identity to form the desired impression in others, with the goal “to manage the impact of stereotypes on others’ perceptions of their competence and character (p. 687).” These IM behaviors are intended to target stereotypes associated with one’s group, thus leveraging positive group stereotypes and counteracting the impact of negative stereotypes. Examples of social-identity based IM behaviors are self-presentational behaviors that involve reducing the salience of a devalued social identity, encouraging others to classify oneself on the basis of personal characteristics, emphasizing similarities with members of more positively valued social groups, or communicating favorable attributes of one’s group. Similar identity management strategies to counteract stereotyping and ultimately reduce workplace discrimination have been proposed by other scholars (Stone and Colella, 1996; Houston and Grandey, 2013; Lindsey et al., 2013; Shih et al., 2013).

One central goal of social-identity based IM consists in refuting negative stereotypes associated with one’s social group (Roberts, 2005; Houston and Grandey, 2013; Lindsey et al., 2013). As older employees are consistently associated with stereotypes of reduced general competence, these stereotypes represent one of the main reasons why their chances of being hired are lower compared to younger employees (Krings et al., 2011). Therefore, engaging in social-identity based IM which consists in proactively refuting negative competence stereotypes, such as being less technology savvy or able to learn, could be a promising strategy for older applicants to ameliorate others’ perceptions of their competence, convey positive impressions and ultimately, increase their likelihood of securing employment. Indeed, older employees are aware of the (negative) stereotypes associated with their group (Finkelstein et al., 2013), and engage in behaviors aimed at distancing themselves from these stereotypes in order to circumvent potential negative outcomes in employment seeking contexts (Lyons et al., 2014).

A crucial question remains: How effective is social-identity IM during the job interview? Does it positively influence decision makers’ perceptions and behaviors toward minority employees? Does it effectively reduce discrimination? To date, there is a serious dearth of research on this topic (Houston and Grandey, 2013). Very little is known about the effectiveness of using social-identity based IM behaviors at selection, and virtually nothing is known about their effectiveness for older applicants. A few studies show that refuting negative stereotypes leads to positive outcomes for minority employees. For example, female leaders were perceived more positively when described as mothers and thus refuting the stereotype of being low in communality (Heilman and Okimoto, 2007). Obese individuals experienced less discrimination from service personnel while shopping, when refuting the stereotype of being weak-willed (King et al., 2006). Two pieces of evidence concerning older applicants examine age discrimination at the pre-interview stage. Bendick et al. (1997) conducted a résumé audit, sending pairs of fictitious applications (one of a younger and one of an older applicant) to 775 firms and employment agencies. In one of the conditions, the older applicant attempted to contradict ageist stereotypes in his letter stating that he is “energetic, adaptable to the latest technology and committed to my career” (p. 41). Results showed that discrimination against this older applicant was still present but reduced by about half. Lahey (2008) conducted a similar study investigating entry-level labor market options for women ages 35–62. Results demonstrated age discrimination, but there was no clear indication that providing statements that counter ageist stereotypes reduced discrimination in pre-interview stages. However, this study focused on female applicants and jobs that suit these applicants only.

Taken together, the majority of the empirical evidence suggests that minority applicants, including older applicants, who use social-identity based IM such as self-promotion to directly contradict common stereotypes associated with their group can diminish stereotypical negative perceptions of them and ultimately increase their chances of obtaining access to employment.

### Current Research

In the present research, we examined the role of social-identity based IM in mitigating employment discrimination against older job applicants. As the theorizing and evidence delineated above suggests, older applicants who employ social-identity IM to contradict the older worker low competence stereotype during the interview will generate more positive or less stereotypical perceptions of competence and hireability and thus, ultimately increase their chances at hiring. Therefore, we expect that older applicants, who engage in self-promotion by providing positive information that runs counter the different facets of the low competence stereotype during the job interview, are perceived as more hirable than those who do not.

*Hypothesis 1*. Older applicants who provide positive information contradicting the older worker stereotype (high IM) will be perceived as more hirable than older applicants who do not (low IM).

Moreover, we examine the extent to which applicant IM behavior does in fact reduce age discrimination by comparing evaluations of both older and younger applicants with equivalent profiles. As suggested by the arguments and evidence presented above, these behaviors should be effective for both younger and older applicants; however, they should be particularly effective for older applicants because they directly target and contradict negative age stereotypes. Therefore, they may ultimately reduce the typically observed hireability gap between older and younger applicants. More specifically, we employed an experimental design that allows comparing evaluations of older and younger applicants under both high and low IM conditions, by fully crossing both factors, applicant age (older versus younger) and IM (high versus low). This design allows determining whether providing positive competence information, such as high IM, has a larger impact on hireability ratings of older compared to younger applicants because, as the theory suggests, only in the case of the older applicants, this behavior implies contradicting the negative stereotype associated with their social group.

*Hypothesis 2*. Engaging in IM reduces age discrimination against older applicants, such that IM will have a stronger positive impact on older than on younger applicants’ perceived hireability.

We addressed our hypotheses using an experimental hiring simulation. Given the lack of extant empirical research for the effectiveness of identity-based IM strategies in reducing discrimination against older applicants during the employment interview, we first conducted a pre-study in which we tested the IM manipulations consequently used in the “Main Study.”

## Pre-Study

The goal of this pre-study was to test the effectiveness of the IM manipulations in five domains of competence subsequently used in the “Main Study.” Importantly, the selected five domains also represent the most common and central facets of the more general older worker low competence stereotype, namely: technology skills, ability to learn, adaptability, ability to handle pressure, achievement orientation (Posthuma and Campion, 2009; Finkelstein et al., 2013). We expected that a high level of IM in a particular competence domain (e.g., adaptability) would lead raters to perceive the applicant as having a higher level of ability in this competence domain (e.g., as being more adaptable), whereas a lower level of IM would lead raters to perceive a lower level of ability in that particular domain.

### Method

#### Participants and Procedure

A total of 150 (104 females, *M*_age_ = 22.00 years, *SD* = 3.03) undergraduate students participated in this study. The duration of the study was approximately 7 min and participation was voluntary.

The study followed a 2 (IM level: low, high) × 5 (age stereotype domain: technology skills, ability to learn, adaptability, ability to handle pressure, achievement orientation) between-subjects design. Participants read a brief excerpt of the interview in which a fictitious male job applicant responded to the interviewer’s questions. The first one was a general introductory question, while the second question asked about the applicant’s level of competence within a specific domain. The applicant’s answer to the first question was identical across conditions, while the answer to the second question varied, depending on the experimental condition, i.e., the particular domain of competence (for details, see “Main Study”). For the IM behavior, we focused on self-promotion, consisting of emphasizing positive qualities and past accomplishments. The IM manipulation was included in the applicant’s answers to the interview questions, such as in the high IM condition, the applicant describes his level of competence in very attractive terms, whereas in the low IM condition he uses more moderate terms. Participants were asked to rate the applicant’s competence in the targeted domain on one item (e.g., “How technologically skilled do you perceive this applicant to be?,” for the technology-skill condition) using a response scale from 1 (not at all) to 5 (very much). At the end, participants provided some demographic information. Note that we did not manipulate applicant age in this study, as it was only designed to assess the effectiveness of the IM manipulations subsequently used in the “Main Study.”

#### Results and Discussion

We conducted five independent-samples *t*-tests to examine the effect of high vs. low IM on perceptions of abilities within each of the five domains. Overall, results indicate that the IM manipulations were effective in positively influencing competence perceptions. That is, when describing their achievement motivation, applicants engaging in high IM were perceived as more achievement oriented (*M* = 4.67, *SD* = 0.48) than those engaging low IM (*M* = 3.60, *SD* = 0.73), *t*(28) = 4.67, *p* < 0.001, *d* = 1.73. When describing their capacity to work under pressure, applicants engaging in high IM were perceived as better able to work under pressure (*M* = 4.27, *SD* = 1.22) than those engaging in low IM (*M* = 2.80, *SD* = 1.08), *t*(28) = 3.47, *p* = 0.002, *d* = 1.27. When describing their capacity to adapt to new situations, applicants engaging in high IM were perceived as more adaptable (*M* = 4.53, *SD* = 0.51) than those engaging in low IM (*M* = 3.27, *SD* = 0.96), *t*(28) = 4.49, *p* < 0.001, *d* = 1.63. When describing their technology skills, applicants engaging in high IM were perceived as more technologically skilled (*M* = 4.33, *SD* = 0.81) than those engaging in low IM (*M* = 2.67, *SD* = 0.90), *t*(28) = 5.31, *p* < 0.001, *d* = 1.93. For learning abilities, the difference between high and low IM did not reach significance, *t*(28) = 1.63, *p* = 0.11; nevertheless, inspection of the means show that applicants engaging in high IM (*M* = 3.93; *SD* = 0.88) were perceived as better able to learn new things than those engaging in low IM (*M* = 3.33; *SD* = 1.11), and that the size of the effect, albeit non-significant, was still of medium size, *d* = 0.59.

Overall, results confirm that our manipulations of IM during the interview influence applicant perceptions within the specific domain as intended: stronger IM leads to more favorable evaluations in the particular domain of competence.

## Main Study

Based on the findings obtained in the pre-study we created a hiring simulation to test our hypotheses. More specifically, participants received a job description and the résumé of a fictitious applicant who was either younger or older. Then, they listened to excerpts of audio recordings of a simulated job interview, enacted by a professional actor who impersonated both the young and the old applicant by using an older and younger sounding voice.

### Method

#### Participants

Participants were undergraduate students recruited at several universities, for a study on interview evaluations. We conducted this study online, with an average duration of the study of approximately 12 min. Participation was voluntary. To further incentivize motivation for the study, participants were informed that they would enter a raffle with the chance of winning one of three gift certificates of 100 Swiss Francs each. A total of 809 participants started the experiment, of which 564 provided complete answers (69% response rate; *M*_age_ = 22.77 years, *SD* = 3.80, 50.53% female, 31.33% employed). After excluding those participants who did not recognize the age of the applicant correctly in a manipulation check (within the age range of 20–29 for the younger applicant, and 50–59 for the older applicant), the final sample consisted of 515 participants (50.29% female, *M*_age_ = 22.87, *SD* = 3.79). About a third of the participants (30.81%) were employed for more than 20 h per week. The demographic composition of the final sample was similar to the one of the initial sample (see above).

#### Procedure

This study followed a 2 (age of the applicant: young, old) × 2 (IM level: high, low) × 5 (age stereotype domain: technology skills, ability to learn, adaptability, ability to handle pressure, achievement orientation) between-subjects design. Participants agreed to complete a study on interview evaluations and were randomly assigned to one of the experimental conditions. They were instructed to assume the role of a hiring manager engaged in a selection process for a travel agent position. This particular job was chosen because the role of a travel agent has been repeatedly demonstrated to be age-neutral, that is, to be perceived as equally suitable for both young and old candidates (Finkelstein et al., 1995; Krings et al., 2011). Also, the travel agent role is a service job involving extensive interpersonal interactions, and therefore the use of self-promotion is natural and may even be an important indicator of competence for the job (Barrick et al., 2010). After providing them with a brief job description, participants reviewed the applicant’s résumé. We manipulated the applicant’s age by indicating his age (29 or 51 years old) and the year in which he obtained his degree (i.e., obtained either 30 or 10 years ago) in the resume and by mentioning the number of years of professional experience (i.e., 20 or 5 years) in the first part of the interview. After reviewing the resume, participants responded to a manipulation check. To disguise the fact that the study was about age, we asked three questions, first about the applicant’s gender, then about his age, and finally about his level of education. Next, they listened to an audio recording of the interview, which contained the same interviewer questions and applicant answers as in the pre-study. For the recordings, we hired a professional actor who enacted both the younger and the older applicant. The actor himself was in his early twenties and he used his normal voice to impersonate the young applicant. To impersonate the older applicant, he modified his voice by imitating typical vocal changes associated with a higher age (e.g., lower pitch, reduced volume, slower speed). The first part of the audio recording was identical across conditions (except for the number of years of professional experience; see below) and it read:

Interviewer: Great, then let’s begin, shall we? I see from your CV that you have vast experience in tourism. Can you tell me a bit about yourself?
Applicant: Hmm….Sure. I started out in Tourism 5/20 years ago, when things were quite different, the internet and social media were less widespread and people relied more on the agencies for planning their travels. I have extensive experience with customers and various computer reservations systems, as emphasized in my resume. My most recent experience was with TCE Travel Agency where I advised customers on wide range of holidays including short haul, long haul, package and bespoke holidays. I am passionate about ensuring that customers have a fabulous experience and what I’m looking for now is a company that values my expertise and where I can have a positive impact on customer relations.

The second part of the interview contained the IM and the stereotype domain manipulations, and thus varied across conditions (see below and Appendix A). As indicated above, age stereotypical domain was manipulated with the five pre-tested interview excerpts (see “Pre-study”) in which the applicant answers the interviewer’s question about his level of competence in one out of five domains (technology skills, ability to learn, adaptability to change, ability to handle pressure, achievement orientation), each referring to a specific facet of competence central to the older worker stereotype (Posthuma and Campion, 2009). IM manipulations were identical to the ones used in the pre-study, such as: In the high IM condition, the applicant engages in higher levels of self-promotion, describing his level of competence in very positive terms whereas in the low IM condition, he uses more moderate terms. Note that the older applicant in the high IM conditions provides information that directly contradicts the older worker low competence stereotype - in other words, he engages in social-identity based IM - whereas the older applicant in the low IM conditions does not. An example of these manipulations is the following (the adaptability to change domain):

Interviewer: Could you also describe me a situation in which a major change occurred in one of your previous jobs and how did you handle it?

##### High IM answer

Applicant: Hmm yeah… for example one situation in which I had to deal with a change was in my previous job when I was suddenly in charge of implementing a new global distribution system. Even though I had vast experience and solid knowledge…I worked with Amadeus and Apollo for more than 4 years… this was by far one of the most challenging tasks…being responsible not only for customer service, but also for budgeting and planning. Plus, assisting my other colleagues transitioning to the new tool. But as difficult as it seemed at first, it was also very stimulating and, dealing with such a change and responsibility has given me the confidence that I can handle other changes and new situations as they occur.

##### Low IM answer

Applicant: Hmm yeah… for example one situation in which I had to deal with a change was in my previous job I was suddenly in charge of implementing a new global distribution system. Even though I had previous experience with GDSes…I worked with Amadeus and Apollo for over 4 years… this was by far one of the most difficult tasks…being responsible not only for customer service, but also for but also for budgeting and planning. Plus assisting my other colleagues transitioning to the new tool. It was difficult and often times stressful, but it gave me valuable experience so in the end I glad I had this challenge.

After listening to the interview excerpts, participants rated the perceived hireability of the applicant and completed a demographic questionnaire.

#### Ethics Statement

This study has been given full clearance by the Ethics Committee of Faculty of Business and Economics of the University of Lausanne and is fully compliant with the Declaration of Helsinki. Respondents were invited to participate in a research study on job interview evaluations. Those who accepted to participate in this study signed an informed consent and then they were asked to review a job application of a male candidate, including a brief job description, a resume, and an excerpt of an audio recorded job interview. Participants then answered a number of survey questions about their evaluation of the candidate as well as a few of demographic questions. Participation was voluntary and participants could withdraw at any time without reprisal. Participation was anonymous and respondents name was not associated with answers. Response were kept completely confidential, to the extent permitted by law. No vulnerable populations were involved in this study.

#### Measures

##### Hireability

We assessed two central facets of perceived hireability, namely person job-fit and hireability. Perceived Person Job-Fit was measured with the three items adapted from Kristof-Brown’s (2000) measure, using a response scale ranging from 1 (not at all) to 5 (very much). Hireability was measured with one item: “Would you hire this applicant for the Travel Agent position?” (1 = *no*, 2 = *rather no*; 3 = *rather yes*; 4 = *yes*). Because all items were strongly correlated (all *r*s > 0.65), we standardized them and created a composite score of hireability (α = 0.88), by averaging responses across the four items.

## Results

To test our hypotheses we conducted a 2 (age of the applicant: old, young) × 2 (IM level: low, high) × 5 (age stereotype domain: technology skills, ability to learn, adaptability, ability to handle pressure, achievement orientation) analysis of variance (ANOVA) with hireability as dependent variable^1^. Results yielded a significant main effect of applicant age, *F*(1,496) = 49.92, *p* < 0.001, $\eta_{p}^{2}$ = 0.091, such that the younger applicant was perceived as being more hireable (*M* = 0.22, *SD* = 0.81) than the older applicants (*M* = -0.23, *SD* = 0.95). This result replicates earlier research by providing supporting evidence for the discrimination against older applicants. Also, a significant main effect of IM emerged, indicating that applicants engaging in high IM, *F*(1,496) = 27.62, *p* < 0.001, $\eta_{p}^{2}$ = 0.053 were perceived as more hireable (*M* = 0.19, *SD* = 0.81) than those engaging in low IM (*M* = -0.18, *SD* = 0.87). This overall effect is true for both younger and older applicants, thus confirming our Hypothesis 1. Moreover, we found a significant main effect of stereotype domain, *F*(1,496) = 4.34, *p* = 0.002, $\eta_{p}^{2}$ = 0.034, indicating that applicants in the achievement orientation condition were rated higher (*M* = 0.14, *SD* = 0.79) than applicants in the learning ability (*M* = 0.08, *SD* = 0.93), ability to handle pressure (*M* = 0.04, *SD* = 0.81), adaptability (*M* = -0.06, *SD* = 0.86) and technology-skills (*M* = -0.21, *SD* = 0.87) conditions. The interaction between applicant age and IM was not significant, *F*(1,496) = 0.72, *p* = 0.396, $\eta_{p}^{2}$ = 0.001. Thus, Hypothesis 2 was not supported across the five stereotype domains. However, we observed a three-way interaction between, applicant age, IM and the stereotype domains that approached significance, *F*(4,496) = 2.03, *p* = 0.089, $\eta_{p}^{2}$ = 0.016. None of the other main effects or interaction terms reached significance, all *F*s ≤ 2.65 and *p*s ≥ 0.10.

To further explore the three-way interaction, we conducted separate ANOVAs with applicant age and IM as factors for each of the five age stereotype domains. Means and standard deviations are presented in **Table 1**.

**Table 1:** Perceived hireability (means and standard deviations using standardized values) of older and younger applicants, using low or high IM, for five age stereotypical domains.

		Low IM				High IM			
		Older applicant		Younger applicant		Older applicant		Younger applicant	
Stereotype domain	*N*	*M*	*SD*	*M*	*SD*	*M*	*SD*	*M*	*SD*
Ability to handle pressure	112	–0.22	0.75	0.03	0.62	–0.04	1.01	0.43	0.68
Learning ability	104	–0.30	0.84	–0.05	1.03	0.26	0.78	0.60	0.82
Achievement orientation	96	–0.15	0.90	0.08	0.74	0.06	0.80	0.58	0.53
Adaptability	102	–0.86	0.82	0.28	0.63	–0.13	0.65	0.40	0.74
Technology skills	101	–0.50	0.77	–0.18	0.96	–0.63	0.68	0.30	0.74

For the ability to handle pressure stereotype and the achievement stereotype domain conditions, the pattern of results was similar. Both ANOVAs yielded a significant main effect of IM, indicating that applicants engaging in high IM were perceived as being more hireable than those engaging in low IM, *F*(1,108) = 3.76, *p* = 0.050, $\eta_{p}^{2}$ = 0.034, and *F*(1,92) = 5.39, *p* = 0.022, $\eta_{p}^{2}$ = 0.055, respectively. Moreover, for both domains, a main effect of applicant age emerged, indicating that older applicants were evaluated less positively than younger applicants, *F*(1,108) = 6.01, *p* = 0.016, $\eta_{p}^{2}$ = 0.053, and *F*(1,92) = 5.86, *p* = 0.017, $\eta_{p}^{2}$ = 0.060, respectively. Thus, for the ability to handle pressure and for achievement orientation Hypothesis 1 was supported, whereas Hypothesis 2 was not.

For the learning ability stereotype domain, only an effect of IM emerged, again showing that applicants engaging in high IM were perceived more hireable than those engaging in low IM, *F*(1,100) = 11.76, *p* = 0.001, $\eta_{p}^{2}$ = 0.105. Therefore, for the learning ability stereotype domain, Hypothesis 1 was supported, but Hypothesis 2 was not.

For the technology-skills condition, we found a significant main effect of applicant age only, *F*(1,98) = 14.55, *p* < 0.001, $\eta_{p}^{2}$ = 0.129, demonstrating that younger applicants were perceived as being more hireable than older applicants. Hence, for this particular domain, Hypotheses 1 and 2 were not supported.

**FIGURE 1:**
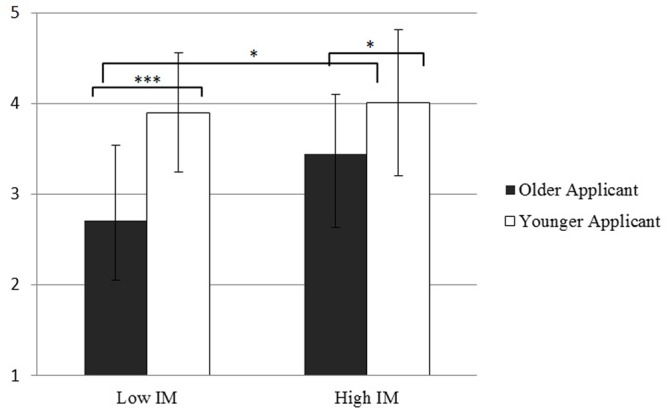
**Perceived hireability (using unstandardized score) of older and younger applicants who use low vs. high IM during the employment interview.** ^∗^*p* < 0.05, ^∗∗∗^*p* < 0.001.

For the adaptability stereotype domain, we found a significant main effect of IM, *F*(1,98) = 8.77, *p* = 0.004, $\eta_{p}^{2}$ = 0.082, showing that displaying high IM leads to more positive ratings than engaging in low IM, which is again in line with our Hypothesis 1, for this particular domain. There was also a significant main effect of applicant age, *F*(1,98) = 35.69, *p* < 0.001, $\eta_{p}^{2}$ = 0.267 showing again that the older applicant was rated lower compared to the younger one. These main effects were further qualified by a significant two-way interaction between applicant age and IM, *F*(1,93) = 4.61, *p* = 0.034, $\eta_{p}^{2}$ = 0.045. The interaction is depicted in **Figure 1** (for illustration purposes, means and standard deviations using unstandardized scores are shown). Follow-up simple main effects analysis showed that engaging in high IM significantly increased perceptions of hireability of the older applicant, *F*(1,98) = 12.80, *p* = 0.001, $\eta_{p}^{2}$ = 0.116, but did not significantly improve those of the younger one, *F*(1,98) = 0.33, *p* = 0.562. This result provides support for Hypothesis 2, showing that for the domain of adaptability, high IM targeting this particular age stereotype is more beneficial for older than for younger applicants. However, results also revealed that despite this benefit, the older applicant was still perceived as less hireable compared to the younger applicant in both the low IM and high IM conditions, *F*(1,98) = 31.73, *p* < 0.001, $\eta_{p}^{2}$ = 0.245 and *F*(1,98) = 7.62, *p* = 0.007, $\eta_{p}^{2}$ = 0.072, respectively.

## Discussion

Although there is a wealth of research exploring the determinants and consequences of discrimination in general and age discrimination in particular, scholars are still far from finding a solution to reduce this still pervasive problem of the modern workplace (Lindsey et al., 2013). The present research aims to come one step closer to an answer, by identifying how IM behaviors can be effectively used by members of stereotyped groups, when they are exposed to potentially discriminatory circumstances. Specifically, we investigated the effectiveness of social-identity based IM strategies in mitigating age discrimination against older job applicants. Social-identity based IM is the process through which individuals actively and strategically influence the impact of stereotypes on other’s perceptions of their personality and competence (Roberts, 2005; Houston and Grandey, 2013). One central element of this form of IM consists in contradicting and thus refuting negative stereotypes, and it has been repeatedly suggested that using these tactics can help reduce workplace bias (Houston and Grandey, 2013; Lindsey et al., 2013; Shih et al., 2013). However, very little is known about the extent to which such IM tactics can reduce discrimination, and even less is known about the extent to which they minimize employment discrimination against older job applicants. This was the starting point of the current study, examining, for the first time, the effectiveness of using identity-based IM to mitigate employment discrimination against older applicants.

The findings of our experimental hiring simulation show that across most of the age stereotypical domains targeted in the interview, applicants who engaged in high IM were perceived as more hireable and a better fit than those who engaged in low IM, which is in line with earlier research that has highlighted the effectiveness of IM for increasing interview ratings (e.g., Gilmore and Ferris, 1989; Tsai et al., 2005). More importantly, our study demonstrates that this effect is also true for older applicants. Thus, older applicants who engaged in IM consisting in contradicting negative competence stereotypes were perceived as more hireable than those who did not. Findings of the pre-study suggest that this effect is – at least partially – due to a the positive impact of the use of self-promotion on perceptions of the applicant’s competence in the targeted domain. Furthermore, results showed that the effect was relatively robust, i.e., older applicants engaging in identity-based IM were perceived as more hireable compared to older applicants who did not engage in such behaviors, in almost all domains of the competence (with the exception of the technology-skills domain, we will come back to this point further in the discussion). Taken together, these findings make an important contribution to the literature because they demonstrate, for the first time, that older applicants can indeed succeed in positively influencing their hiring chances when they actively counteract negative age stereotypes during the job interview. The fact that the overall positive impact of IM on older applicants’ hireability ratings was true across several facets of competence associated with the most common age stereotypes is particularly encouraging.

However, supporting evidence for the contention that using such IM tactics helps *overcome* discrimination was scarce. While engaging in high IM consistently boosted older applicants’ hireability ratings, they were still perceived as less hireable than younger applicants, when displaying the same type of IM. Thus, despite the positive effect of IM on older applicants’ hireability, overall age discrimination persisted and the proposition that IM eliminates it was not supported. However, for one out of the five stereotype domains (i.e., for adaptability), we did find evidence that using IM tactics is particularly beneficial for older compared to younger applicants. This result is noteworthy because reduced adaptability has been identified as one of the primary dimensions of the older worker stereotype, together with incompetence and warmth (Marcus et al., 2016). Therefore, actively contradicting adaptability stereotypes can be a viable strategy for older applicants to influence recruiters’ impressions.

We can only speculate why this beneficial effect was restricted to the adaptability stereotype and why it did not emerge for the other domains. Two explanations come to mind: first, it is possible that adaptability was more pertinent for the travel agent job, compared to the other stereotype domains (e.g., ability to work under pressure). Indeed, as indicated by O^∗^net the travel agent role requires a high degree of direct interaction with clients and also peers, therefore the ability to adapt to each individual client to best meet their needs represents a major part of the role. Results of a brief survey confirm this assumption, indicating that adaptability is in fact perceived as a crucial competence for the travel agent role.^2^ As a consequence, contradicting the older worker low adaptability stereotype may have been particularly important in this context and hence, effective in influencing subsquent hireability ratings. Second, it is possible that for some domains of competence, older applicants engaging in high IM appeared less credible or authentic than their younger counterparts when describing their strong skills and abilities. This may have raised suspicion regarding the older applicant’s truthfulness. It may also have undermined participants’ belief that they can easily “read” the applicant and distinguish truth from fiction in the applicant’s responses, which has been shown to influence the favorability of interview outcomes (Roulin et al., 2014). Thus, it is possible that for some domains of competence, the effectiveness of IM in boosting hireability ratings was reduced for the older applicant because they appeared less credible. This opens the possibility that IM may even backfire for older applicants. Indeed, Houston and Grandey (2013) caution about the potential backfire of using IM strategies aimed at refuting stereotypes, arguing that such tactics may in fact increase the salience of stigma and/or highlight the deficiencies members of stereotyped groups are trying to overcome. The potential to backfire might be particularly increased when using self-promotion tactics, as suggested by the “self-promoter paradox” (Jones and Pittman, 1982). This paradox contends that overt claims about one’s competence may lead the perceiver to assume that the applicant might try to compensate, or even cover up, for a lack of competence.

Taken altogether, the results of the current research show that using IM tactics, rooted in social identity during the employment interview, is indeed beneficial for older applicants, as they positively influence perceptions of hireability for older applicants. However, promising this finding, we also found that discrimination against older applicants was still present, despite the positive impact of such IM tactics. In fact, overall, we found little evidence for the contention that using these tactics helps eradicate discrimination. However, we believe that it is premature to consider them ineffective because employment discrimination is a robust and complex phenomenon (Dipboye and Collela, 2005). It is possible that it takes more than providing counter-stereotypical information during the interview to eliminate it. In this research we focused on verbal self-promotion IM behaviors and hence we did not include other forms of IM tactics. As previously demonstrated the combination of self-focused IM strategies together with other-focused IM strategies (e.g., ingratiation, opinion conformity) seems more effective for increasing perceptions of competence and fit (Proost et al., 2010) than the use of various IM tactics alone. Similarly, past research has shown that non-verbal behaviors, in particular smiling, eye contact, nodding or hand gesturing are consistently associated with higher interview evaluations (Burnett and Motowidlo, 1998; Peeters and Lievens, 2006). Perhaps, verbal social-identity based IM strategies are only effective in eradicating discrimination if they are corroborated with non-verbal IM tactics aimed at eliciting likability. Thus, it may take the combination of tactics to successfully eliminate discrimination.

### Study Limitations and Future Research

While our study makes substantive contributions to the existing literature, it has some limitations. A first limitation is related to the methodological design. We chose a controlled between-subject experimental design, to isolate the effects of applicant age and use of IM tactics on hireability evaluations. While this methodological approach was necessary to establish the effectiveness of IM strategies in mitigating age discrimination, it remains somewhat artificial. Participants listened to an extract from a previously conducted job interview, and reviewed only one applicant. While we made considerable efforts to ensure the realism of our study, its external validity is somewhat limited because in a real interview context, interviewers have a more dynamic role, being able to ask additional clarification questions and to assess several competencies before making a recommendation for hiring. Moreover, the length of the interview (∼3 min) may have appeared somewhat artificial and potentially reduce raters’ accountability. While this raises legitimate concerns, short interviews are not uncommon in practice and evidence has shown that often the initial impressions formed during the first seconds of the interview tend to persist during the interaction and even determine the final outcomes (Curhan and Pentland, 2007). Nevertheless, given the short duration of the interview, it is possible that the IM behavior applied in our study was more salient than it would have been in a longer interview. Hence, it remains an open question for future research to examine how repeated incidences of this type of IM behavior might influence outcomes in longer interviews. Finally, during typical selection processes, several candidates are evaluated against each other. Therefore, future research should also examine whether our findings can be replicated by using a within-subjects design where actual recruiters evaluate several applicants in full length interviews.

Our main study was conducted with undergraduate students. While about a third of the participants had substantial work experience, the remaining two thirds did not. It is possible that their lack of professional experience as well as their relative young age has influenced their behavior during the study, suggesting that future studies should aim to replicate our results, using a more age-diverse sample with more professional experience. Ample evidence shows, however, that this is not a concern when studying discrimination. As previous studies revealed, that students and professional decision makers react in the same fashion, such as they are similarly susceptible to social discrimination (e.g., Olian et al., 1988; Hosoda et al., 2003). Very similar behaviors by students and professional decision makers have also been shown in the field of age discrimination at employment (Krings et al., 2011; Kaufmann et al., 2015).

Finally, we examined the effects of identity-based IM in five facets that are central elements of the older worker stereotype because we wanted to cover the main elements of the older worker stereotype (Posthuma and Campion, 2009). However, it is possible that these competencies are differentially related to the specific requirements of the travel agent role. Interaction with the public, including customers, service providers, collaborators, and peers, is a major part of the travel agent’s activity, as well as the use of computers and technologies to assist beneficiaries. Indeed, several pieces of evidence suggest that adaptability is a highly important competency for the travel agent role. Therefore, we speculated that the use of IM in the adaptability stereotype domain was particularly beneficial for the older, compared to the younger applicant because this domain is highly relevant for the job. Future research could account for a better match between the precise responsibilities necessary for the job and the stereotype domains covered during the interview.

### Implications

This study has several implications for both research and practice. An important theoretical implication addresses the need for more research regarding the effectiveness of IM strategies to counteract negative stereotypes and combat employment discrimination (Houston and Grandey, 2013). Using models of IM and social identity, we show that providing counter-stereotypical information does enhance hireability evaluations of minority – in our case, older applicants; however, more research is needed to understand which circumstances facilitate this mechanism and to what extent their impact increases, decreases or even becomes hurtful. As previously noted, this mechanism may be most effective and beneficial when it focuses on the most central competency required by the job. It may be less effective for more peripheral competencies. And it may even backfire, when members of negatively stereotyped groups would be perceived as overtly trying to overcome competence deficiencies (Houston and Grandey, 2013). It is thus important to identify under which conditions engaging in social-identity based IM is most effective and has a positive impact, and under which conditions the same behavior may actually have little effect or even bear the potential to backfire and hurt minority applicants’ access to employment.

From a practical perspective, our results offer a viable solution to individual applicants to deal with prejudicial and discriminatory treatment against them. For example, integrating self-promotion tactics aimed at refuting negative stereotypes in future interview training programs could help older job seekers capitalize on their strengths (Kristof-Brown et al., 2002). However, salutary these strategies may seem, it is important to note that we do not imply that efforts to combat employment discrimination pertain solely to the individual applicants. On the contrary, organizations must continue to strive for ensuring an age-diverse workforce and create equal employment opportunities for both younger and older employees. Therefore, implementing measures such as standardized interviews or rater training programs (e.g., Roch et al., 2012) that help reduce bias and improve rater accuracy are valuable venues for organizations and have been shown to mitigate subgroup differences in interview ratings (Huffcutt et al., 2001). Moreover, as Boehm et al. (2014) demonstrate, fostering age-inclusive HR practices contribute not only to an age-inclusive organizational climate, but also they also increase firm performance and reduce turnover intentions. Also, it is plausible to assume that identity-based IM tactics are less likely to backfire under age-inclusive organizational climate conditions. Ultimately though, if organizations are successful in establishing and reinforcing an age-inclusive organizational climate, it should be less necessary for older applicants to make use these IM tactics.

## Conclusion

This study emphasizes the role played by applicants’ use of social-identity based IM strategies in reducing employment discrimination against older employees. Our findings show that while using such strategies during the interview improves evaluations of older applicants, older applicants continue to be evaluated as less hirable than their younger counterparts. Thus, while such tactics do have a positive impact on how older applicants are perceived at employment, and hence demonstrate their *potential* for mitigating discriminatory treatment, their effectiveness for *eliminating* discrimination seems to be insufficient. Given the complexity of the issue, further research is needed to identify the conditions under which these tactics reach their full potential, i.e., they become able to fully eradicate discrimination.

## Author Contributions

Both authors contributed equally to the following tasks: Development of the idea and of the methodology, data analyses and interpretation, writing of all sections of the manuscript. IG collected the data.

## Conflict of Interest Statement

The authors declare that the research was conducted in the absence of any commercial or financial relationships that could be construed as a potential conflict of interest.

## Footnotes

1 Preliminary analyses showed no significant effect of rater age, *F*(1,480) = 1.89, *p* = 0.169. Therefore, age was not included in the main analyses.

2 A survey was conducted on a comparable sample with the one used in the main study (*N* = 20 undergraduate students, *M*_age_ = 23.15 years, *SD* = 2.77, 60% female). Results indicate that adaptability was considered the most important competence for the travel agent role, followed by ability to handle pressure, technology skills, learning abilities, and achievement orientation.
