# Supplementary material for: Impression Management in the Job Interview: An Effective Way of Mitigating Discrimination against Older Applicants?
Source: Front Psychol. 2017 May 16;8:770. doi: 10.3389/fpsyg.2017.00770 (PMC5432631; doi:10.3389/fpsyg.2017.00770)
Supplement: Supplementary file 1 [file Data_Sheet_1.DOCX]

APPENDIX A

**Question – Answers: Introduction (identical across conditions)**

Interviewer: Great, then let’s begin, shall we? I see from your CV that you have vast experience in tourism. Can you tell me a bit about yourself?

Candidate: Hmm….Sure. I started out in Tourism 5/20 years ago, when things were quite different, the internet and social media were less widespread and people relied more on the agencies for planning their travels. I have extensive experience with customers and various computer reservations systems, as emphasized in my resume. My most recent experience was with TCE Travel Agency where I advised customers on wide range of holidays including short haul, long haul, package and bespoke holidays. I am passionate about ensuring that customers have a fabulous experience and what I'm looking for now is a company that values my expertise and where I can have a positive impact on customer relations.

**Question-Answer: Ability to Handle Pressure Condition**

Interviewer:  How well do you deal with stress and working under pressure?

***High IM Answer:***

Candidate: I believe *I manage* pressure very well. In my previous job, I often had to finish tasks under short deadlines and actually I have to say I prefer it in several ways. As a travel agent you constantly deal with customers and there was a lot of pressure to address customers’ queries while also completing administrative tasks. *When I work under pressure I tend to be more focused and motivated. It keeps me alert and helps me complete my tasks on time and effectively. I must say this kind of drive, keeps me going.*

***Low IM Answer:***

Candidate: I believe *I can manage* pressure well. In my previous job, I often had to finish tasks under short deadlines, so I am used to working under pressure. As a travel you constantly deal with customers and there is a lot of pressure to address customers’ queries while also completing administrative tasks. *While I generally prefer to take my time and do the task thoroughly to avoid mistakes, I understand pressure is sometimes necessary*.

**Question-Answer: Learning Ability Condition**

Interviewer: The next question is about how you learn new things. How open are you to learning new e-travel applications or procedures for helping customers? Can you give me an example of a situation when you learned something new?

***High IM Answer:***

Candidate: *Actually, I’m quite open, learning new things comes easily to me*. For example, in my last job I had to learn how to use the company's in-house web application to track the status of my sales and the best travel deals for the region of the world I was acting as an agent for. *It was hard at first because the application was not very user friendly but I enjoyed the challenge and soon I found I was able to use the application's algorithms to do tracking that even my colleagues weren't sure how to do*.

***Low IM Answer:***

Candidate: *Sure. I am open to learning new things and I think I’m good at it.* One example? Yeah, in my last job I had to learn how to use the company’s in-house web application to track the status of my sales and the best travel deals for the region of the world I was acting as an agent for. *It was very difficult at first because the application was not very user friendly and I had to invest a lot of time familiarizing myself with it. It was a challenge but after a while I was able to use it as well as the rest of my colleagues.*

**Question-Answer: Achievement Orientation Condition**

Interviewer: Where do you see yourself in 5 years?

***High IM Answer:***

Candidate: Hmm…I see myself five years from now being in a *managerial position.* *Though it is quite ambitious I believe that with hard work and a well set plan, it can be achieved. I see myself progressing, learning new skills, and having more responsibilities. My goal is to improve clients’ experiences while contributing to the development of the company. I can see many challenges lying ahead of me, but I am eager to experience them.*

***Low IM Answer:***

Candidate: Hmm…I see myself five years from now being in a *good position, where people can recognize me as a good employee and as an asset of their organization. Although the future is unpredictable, what I can say for sure is that the day I will join the firm, I will try to understand my role in the company, and will give my 100% to make my work productive and whenever to contribute fully to the development of the organization.*

**Question-Answer: Adaptability Skills Condition**

Interviewer:  Could you also describe me a situation in which a major change occurred in one of your previous jobs and how did you handle it?

***High IM Answer:***

Candidate: Hmm yeah… for example one situation in which I had to deal with a change was in my previous job when I was suddenly in charge of implementing a new global distribution system. Even though I had *vast experience and solid knowledge*…I worked with Amadeus and Apollo for more than 4 years… this was by far one of the *most challenging* tasks…being responsible not only for customer service, but also for budgeting and planning. Plus, assisting my other colleagues transitioning to the new tool. *But as difficult as it seemed at first, it was also very stimulating and, dealing with such a change and responsibility has given me the confidence that I can handle other changes and new situations as they occur*.

***Low IM Answer:***

Candidate: Hmm yeah… for example one situation in which I had to deal with a change was in my previous job I was suddenly in charge of implementing a new global distribution system. Even though *I had previous experience* with GDSes…I worked with Amadeus and Apollo for over 4 years… this was by far one of the *most difficult* tasks…being responsible not only for customer service, but also for but also for budgeting and planning. Plus assisting my other colleagues transitioning to the new tool. *It was difficult and often times stressful, but it gave me valuable experience so in the end I glad I had this challenge.*

**Question-Answer: Technology Skills Condition**

Interviewer: So, as you’ve seen in the job description this position requires using e-travel applications and social media. How familiar are you with computers to help customers find the best deals?

***High IM Answer:***

Candidate: Well… *I am quite technologically savvy*. *I have taken several computer classes, as you can see from my CV, I am familiar with both Windows and* *Mac operating systems and I am also pretty good at troubleshooting*. *Also, I am experienced with the MS Office (Word, Excel, Power point) and also Prezi and Editor. I’m also very much into social media, I’m actively using Facebook and Twitter. My teenage nephew was making fun of me for posting and twitting more than he was, haha. I’m kind of a young spirit when it comes to new technologies; haha.*

***Low IM Answer:***

Candidate: Well… *I am familiar with computers*. *I use email, Outlook for work and Yahoo for my personal email and of course Word, Excel, and Powerpoint. I could say I am quite competent in browsing on the Internet, doing online transactions, booking flights and hotels, all these things necessary for the job. But I have to say when it comes to social media like Facebook or Twitter and other new applications I am not very up to date, like the young generation is. Last year my teenage nephew set me up a personal Facebook account but I didn’t really get into it and I think I’ve already forgotten my password (laughing). I guess I’m too old for these things, haha.*
